# Increased prevalence of obesity, diabetes mellitus and hypertension with associated risk factors in a mine-based workforce, Democratic Republic of Congo

**DOI:** 10.11604/pamj.2019.34.135.20226

**Published:** 2019-11-07

**Authors:** Paul Makan Mawaw, Thierry Yav, Olivier Mukuku, Olivier Lukanka, Patrick Mumba Kazadi, Daniel Tambwe, Jules Omba, Jean-Baptiste Kakoma, Michael John Bangs, Oscar Numbi Luboya

**Affiliations:** 1 Department of Public Health, University of Lubumbashi, Democratic Republic of Congo; 2 Department of Research, Institut Supérieur des Techniques Médicales de Lubumbashi, Democratic Republic of Congo; 3 Occupational Health Clinic, International SOS, Tenke Fungurume Mine, Democratic Republic of Congo; 4 International SOS Clinic, Kinshasa, Democratic Republic of Congo; 5 Anglo-Gold Ashanti, Mongbwalu Clinic, Democratic Republic of Congo; 6 Department of Gynecology and Obstetrics, University of Lubumbashi, Democratic Republic of Congo; 7 PT Freeport Indonesia/International SOS Public Health and Malaria Control, Kuala Kencana, Papua, Indonesia

**Keywords:** Non-communicable diseases, mine workforce health, obesity, diabetes, hypertension, Democratic Republic of Congo

## Abstract

**Introduction:**

The burden of non-communicable diseases (NCDs) is increasing rapidly in low- and middle-income countries, with the largest portion occurring in Africa. Results from earlier baseline measures on obesity, diabetes and hypertension (ODH) in the Tenke Fungurume Mining (TFM) workforce in 2010 showed high proportions of overweight, pre-diabetic and pre-hypertensive individuals, predicting an upward trend in the burden of ODH over time. The 2010-2015 longitudinal trends on ODH and related risk factors among the TFM workforce is presented herein, and projects the consequent burden of these diseases on the workforce by 2025 if an effective prevention program is not implemented.

**Methods:**

A longitudinal, retrospective cohort study with 3-time intervals was conducted using occupational health records collected on all employees and contractors who had a pre-employment or follow up medical checkups covering the period between January 2010 and December 2015. Repeated paired t tests measured changes in mean values of quantitative risk factors, while a chi-square test assessed changes in prevalence and categorical risk factors over time. A linear projection model was used to predict the consequent morbidity of ODH for the subsequent 10 years up to 2025.

**Results:**

Between 2010 and 2015, prevalence increased from 4.5% to 11.1% for obesity, 11.9% to 15.6% for diabetes, and 18.2% to 26.5% for hypertension. By 2025, provided no prevention program is implemented, prevalence is predicted to reach 25%, 24% and 42% respectively for obesity, diabetes and hypertension.

**Conclusion:**

Without implementation of a comprehensive NCD prevention plan, the burden of ODH and other NCDs is predicted to increase dramatically in the TFM workforce. Alone or combined, NCDs have the potential to dramatically increase operational costs while decreasing productivity over time.

## Introduction

Collectively, Non-Communicable Diseases (NCDs) are the leading cause of premature death globally. In 2015, they contributed to an estimated 39.5 million deaths, approximately 70% of total mortality worldwide, of which 15 million deaths occurred below the age of 70 years [1]. Nearly 80% of these deaths occurred in designated Low- and Middle-Income Countries (LMICs). Combined, NCDs represent the major health and development challenges of today, both in terms of direct human suffering and burdens on the socioeconomic fabric in all societies. In particular, the overall disabling effect on economically productive adults, those who bear enormous social responsibilities in communities, is tremendous [1-5]. The NCD burden is increasing rapidly in LMICs, the largest portion (24%) occurring in Africa [6]. Globally, Cardiovascular Diseases (CVD), cancers, chronic respiratory diseases, and Diabetes Mellitus (DM) are the four primary NCDs linked with common attributable risk factors. Aggregated, they represent the leading cause of death and are responsible for around 82% of all NCD-related mortality [1,3]. The leading killer among NCDs is CVD that accounted for approximately 17.7 million or 46.2% of all NCD-related deaths in 2014 [4].

The economic burden of CVD in Africa is increasing [7], expected to cost the continent billions of dollars in excess productivity loss and expenditures in the decades to come. The financial burden comes in the form of direct healthcare costs for the treatment of CVD and controlling risk factors that contribute to disease. These costs are borne by individuals, families, communities, governments, and the private sector; they include decreased worker productivity due to absenteeism or premature death, and the loss of vital savings and assets when families face major healthcare expenditures such as assisting with prolonged rehabilitation following stroke or repeated dialysis after renal failure [8]. Obesity, diabetes mellitus and hypertension (ODH) are the major risk factors for CVD [9,10]. Globally, the proportion of disease attributable to hypertension has increased significantly from 4.5% in 2000 [10], to 7% in 2010 [11]. Hypertension is estimated to cause 7.5 million deaths, credited with around 12.8% of the global all-cause mortality [12]. The prevalence of hypertension in Africa in 2008, approximately 46% of the entire adult population, was the highest in the world, a condition representing the leading CVD risk factor in sub-Saharan Africa [12,13]. The World Health Organization (WHO) identified hypertension as the greatest NCD problem in the Democratic Republic of the Congo (DRC) in 2014, with one of the highest prevalence rates in Africa (24.8% of adults) [4]. Results from the baseline cross-sectional study on ODH in the Tenke Fungurume Mining (TFM) workforce in 2010 showed a hypertension prevalence of 18.2% [14]. Hypertension is a leading cause of significant financial stress on families, including the cost of care for medical-related complications arising from the consequences of stroke, ischemic heart disease, and congestive heart failure [8].

Overweight and obesity, defined as a body mass index (BMI) of ≥ 25.0 kg/m² and ≥ 30.0 kg/m², respectively, were linked to 3.4 million deaths globally in 2010 [11]. From 2010 to 2014, the prevalence of overweight in adults aged 18 and over in the DRC increased from 18.8 to 20.6%, while obesity rates rose from 3.7 to 4.4% [4]. Results from the 2010 baseline study on ODH in the TFM workforce found a BMI obesity prevalence of 4.5% [14]. With current trends, by 2030, the number of diabetics is expected to reach 439 million globally [15]. This increase will be more marked in developing countries where the estimated number of diabetics will rise dramatically from 84 million to 228 million [16]. Between 1980 and 2014, the estimated number of diabetics in adults aged 18 or more years in the African region increased from 7 million to 25 million, with an estimated prevalence of 7.1% in 2014 [17]. In the DRC, diabetes mellitus has increased steadily from 5.7% to 6.1% between 2010 and 2014; whereas 11.7% of the TFM workforce was clinically diabetic in 2010 [4,14]. Findings from the TFM baseline data are highly suggestive of the significant burden of ODH and their associative risk factors among mining workers. Additionally, the proportions of pre-obese (19.7%), pre-diabetic (16.5%), and pre-hypertensive individuals (47.8%) indicated that without significant interventions to curb the epidemic, a significant increase in the prevalence of ODH in this population would occur over time [14]. This study describes longitudinal trends of ODH prevalence and their related risk factors among the TFM workforce between 2010 and 2015 and projects on the consequent burden of ODH by 2025 provided effective prevention programs are not implemented.

## Methods

**Figure 1:**
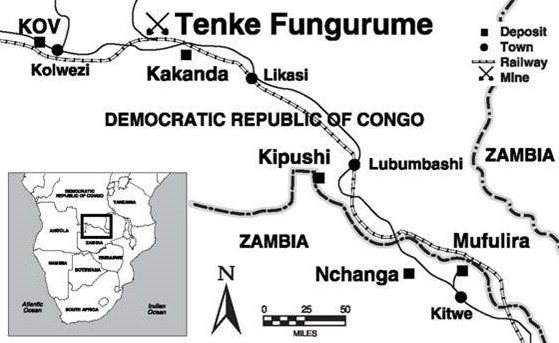
Tenke Fungurume Mine location, DRC (Source: Freeport-McMoRan Inc.)

**Study site and population:** this study is part of an ongoing occupational health project on NCDs among a mining workforce in southern DRC. The mining site near the townships of Tenke and Fungurume is located approximately 90 km southeast of Kolwezi in Lualaba Province (Figure 1). Tenke Fungurume Mining (TFM) operates one of the largest copper and cobalt extraction and production sites in the DRC and is one of primary employers in the province. The study population included all workers aged 20 years and greater, employed either directly by the mining company or its affiliated contractors, who had undergone a mandatory Occupational Medical Check-Up (OMCU) during the initial recruitment phase and had subsequent annual follow-up OMCU at the onsite Occupational Health Clinic (OHC) between January 2010 through December 2015. Collected employee medical data is retained in hard copy forms at the site OHC. During the observation period, the mining company employed approximately 8,000 people, of which males made up 94% of the workforce, 74% below the age of 45 years, and approximately 98% representing Congolese nationals. Field workers made up 69% of the total workforce; typically working in routine timed shifts of either 8 h or 12 h intervals and often involving varying periods of vigorous physical activity. Non-shift office workers comprise the remaining 31% of the permanent workforce, those spending the majority of their working time sitting while doing routine desk-bound activities involving relatively minimal physical activity throughout the day. Regular meals are provided by the employer free-of-charge to employees residing in the residential camps three times daily (breakfast, lunch pack and evening meal) and on fixed scheduled periods (excluding the lunchtime meal pack). Excluding those workers living nearby outside the mine residential camps, few employees have access (or need) for significant external sources of nutritional intake.

**Study design and data collection:** the study design was a retrospective follow-up study using a historical cohort comparing risk factors to outcomes over multiple years. Data were collected from existing medical records using a non-randomized approach provided individuals met study eligibility criteria. This longitudinal study used time series data linked to the mine workforce, stored as hard copies at the onsite OHC. Entry-level study inclusion criteria demanded that only mine workers who had underwent pre-employment and/ or had subsequent routine standard annual OMCUs during the period between January 2010 and December 2015 is included in the analysis. Those with a 2010 “baseline” (T0) measure (entry-level or routine) but lacking complete follow-up medical examination entries during the follow-up 5-year period (T1- T5) were excluded in the analysis. Data were transcribed and collated for three time points: Baseline, defined as starting point OMCU (either by pre-employment or standard annual medical assessment) in 2010, followed by an OMCU at year-3 (2013), and year-5 (2015). Study participants' occupational health files were carefully reviewed in June 2018 with the following information collected on separate standardized study forms:

**Sociodemographic and occupational characteristics:** gender, age, nationality, permanent residence at work site, occupational work grade and potential hazard exposure history (dusts, vapors, smoke, chemicals, e.g., industrial solvents, acids, bases, sulfur, paint, hydrocarbons; excess noise, e.g., a work environment requiring hearing protection, and whole-body vibration).

**Anthropometric and medical parameters:** weight, height, systolic and diastolic blood pressure, fasting blood glucose, total cholesterol, evidence of hypertension, diabetes, and heart disease.

**Behavioral characteristics:** self-reported status as a current smoker and amount of daily alcohol use. As part of pre-employment and subsequent annual OMCUs, all screened participants underwent a standardized physical examination including calibrated measurements of height, weight, and blood pressure (at the midpoint of the arm, with an electronic blood pressure monitor (Welch Allyn^®^ New York, USA, Ser. No. (21)20160108472). Blood samples were obtained for measuring total serum cholesterol and overnight (12h) fasting plasma glucose using calibrated laboratory-based analyzers (CYPRESS Cyanexpert130^®^, CYPRESS, SN026CD01/2015). Based on these measurements at time of each MCU, metabolic risk factors for NCDs were defined and assessed based on 2011 WHO criteria [18].

**Overweight & obesity:** defined aged ≥ 20 years with a body mass index (BMI) ≥ 25.0 kg/m^2^, where BMI is calculated as weight in kilograms divided by the square of body height in meters. Obesity is defined as individuals with BMI ≥ 30.0 kg/m^2^. Overweight and obesity prevalence is the proportion of the total samples with BMIs as defined above.

**Elevated blood glucose:** defined aged ≥ 20 years with a fasting blood glucose ≥ 126 mg/dl. Diabetes prevalence is defined as the proportion of individuals with a fasting glucose ≥ 126 mg/dl or with known diabetic condition with blood glucose lower than 126 mg/dl and under hypoglycemic therapy.

**Elevated cholesterol:** defined aged ≥ 25 years with total serum cholesterol ≥ 190 mg/dl. Prevalence of hypercholesterolemia was defined as the proportion of individuals with total serum cholesterol ≥190 mg/dl, or known history of hypercholesterolemia with normal serum total cholesterol level while taking anti-cholesterol management medication.

**Elevated blood pressure:** defined aged ≥ 20 years with systolic blood pressure ≥ 140 mm Hg and/or diastolic blood pressure ≥ 90 mm Hg. Prevalence of hypertension was defined as the proportion of individuals with a systolic blood pressure (SBP) ≥ 140 mmHg and/or a diastolic blood pressure (DBP) ≥ 90 mmHg, or known history of hypertensive condition with normal SBP and/or DBP while taking antihypertensive medication.

**Smoking and alcohol use:** alcohol consumption was based on self-reported use by employee and entered in the Occupational Health examination form. The consumption of four or more “standard units” of alcohol per day was the cut-off measure between “low/normal” and “high” alcohol use. One standard “unit” is the equivalent 10 ml (~8 g) pure ethyl alcohol. Depending on the amount consumed and percentage alcohol per volume, a typical alcoholic drink might contain between 1-3 units. For analysis, smoking behavior was restricted to individuals who indicated they smoked tobacco every day at the time of the OMCU. Cigarette use was divided between those reporting having smoked on average less than 10 cigarettes and those who smoked ≥ 10 per day.

**Data analysis:** Data points between T0, T3 and T5 were based on calendar year periods, not individual person-time units between OMCUs. Data were transcribed in Epi Info^TM^ 7.1.4.0 (CDC, Atlanta, GA, USA) and aggregated and analyzed using Stata^®^ 14 (Stata Corp LLC, College Station, TX, USA). Data analysis was performed using primarily descriptive statistics. The Fisher's exact test (2-sided) was used to examine differences in exposures and outcomes between male and females. The McNemar chi-squared test assessed changes in prevalence of ODH and categorical risk indicators for each time increment in years from baseline (i.e., from T0 to T3 and T0 to T5). Repeated paired Student's t-tests was preformed to assess change in mean values of quantitative risk indicators. The delta (Δ) change was defined as the difference between mean values of quantitative risk indicators at two different points in time. For projection on consequent prevalence (morbidity) of ODH by 2025, a linear model was used (y= ax+b), where y is the projected prevalence, x is time, b is the baseline prevalence (the value of y when x=0), and is the slope of the line [i.e., the increase in prevalence (y) for a unit increase in time (x)]. The relative risk (RR) determined the association between professional work grade, nature of work (field- or office-based), known occupational exposures/ hazard (noise, vibrations, and chemical exposure) and the occurrence of ODH in employees.

**Ethical considerations:** Before beginning the study, ethical review and approval was obtained from the Medical Ethics Committee of the University of Lubumbashi, DRC. All employee medical information used in this study was obtained through standardized pre-employment and/or routine annual health examinations conducted at the same OHC. Qualified medical and paramedical staff transcribed designated parameters from original records. No additional biological samples were collected for analysis, nor were employees interviewed. Given the nature of the study, it was not possible or deemed necessary to obtain informed consent retrospectively from participants. Pre-employment occupational health medicals and annual OMCUs are TFM employment requirements. All data collected and used in the analysis were treated anonymously, held under strict confidentiality ensuring no personal information (identifiers) was disclosed at any time to those not directly involved in the study.

## Results

Occupational health files of mining company employees and contractors were carefully reviewed to identify those that underwent mandatory pre-employment health examination and/ or annual follow-up OMCUs between calendar years 2010 and 2015. In total, medical data were available for 2,749 workers (2,574 men, 175 women) at baseline (T0), 4,751 workers (4,448 men, 303 women) at T3 (2013)-4, and 5,015 (4,697 men, 318 women) at T5 (2015) MCU monitoring periods. However, for longitudinal analysis, the study only included files of employee subjects who had a complete medical follow-up between 2010 through 2015 covering the three data collection points of interest. After screening, 2,715 employee files comprising 2,544 males and 171 females were used for data collation and analysis. Demographic, behavioral and occupational characteristics of study subjects at T0, T3, and T5 follow-up periods are presented in Table 1. The percentage of those aged 45 years or older was 23.4% at T0, 31.7% at T3, and 38.6% at T5 follow-up times. Compared to males, females were younger in mean age and more than 50% held an office/clerical position of employment. Females represented 6.3% of the study cohort and near identical with the overall proportion of total female employees having undergone at least one OMCU during the 6-year period. The proportion of all subjects who reported one or more occupational exposure/ hazard was 21.9 % from T0 to T5. Subjects reporting exposure to one or more key physical hazards (noise and/or vibration) remain consistent at 48.2% from baseline to T5. The percentage reporting to smoke (regardless the amount or frequency) were 19.0%, 18.6% and 18.2% at T0, T3, and T5, respectively; while alcohol use remained consistent at between 40.1 and 41.6% from baseline to end of observation. Table 2 provides successive key anthropometric, medical and metabolic characteristics of study subjects during the observation period. Underweight subjects (BMI <18.5 kg/m^2^) decreased from 6.5% at baseline, to 1.8% at T3, and 1.0 at T5, conversely the proportion of obese people more than doubled from baseline (4.4%) to T5 (11.1%). The percentage of the cohort with normal weight at baseline (69.3%) decreased in favor of becoming overweight (BMI 25-29.9) over time. Overweight individuals represented 19.8% at T0 to 32.8% at T5. The percentage of those with elevated blood sugar (≥ 126mg/dl) increased from 5.7% at T0, to 7.6% at T3 to 8.3% by T5, while hypercholesterolemia was seen in 8.4% of study subjects at baseline, 13.6% at T3, and 14.3% at T5. The percentage of people with elevated SBP had increased significantly, more than doubling between baseline (8.6%) and T5 (23.9%). For individuals with elevated DBP, the percentage has increased from 10.1% at baseline, to 19.9% at T5.

**Table 1:** Study characteristics of employees at baseline T0, T3 and T5 medical follow-up monitoring periods

Variables		T0, 2010)			T3, 2013)			T5, 2015)		
		Total (N = 2715)	Males (n = 2544)	Females (n = 171)	Total (N = 2715)	Males (n = 2544)	Females (n = 171)	Total (N = 2715)	Males (n = 2544)	Females (n = 171)
**Age (years) Mean (SD)**		38.0 (8.9)	38.0 (9.0)	36.0 (8.3)	40.0 (8.9)	40.9 (8.9)	39.0 (8.3)	42.9 (8.9)	43.0 (8.9)	41.0 (8.3)
**Age range (years)**	18-29	483 (17.8%)	445 (17.5%)	38 (22.2%)	196 (7.2%)	175 (6.9%)	21 (12.3%)	92 (6.4%)	86 (3.4%)	6 (3.5%)
	30-34	649 (23.9%)	605 (23.8%)	44 (25.7%)	546 (20.1%)	509 (20.0%)	37 (21.6%)	391 (14.4%)	359 (16.8%)	32 (18.7%)
	35-39	534 (19.7%)	493 (19.4%)	41 (24.0%)	633 (20.3%)	589 (23.6%)	44 (25.7%)	649 (23.9%)	605 (25.4%)	44 (25.7%)
	40-44	413 (15.2%)	392 (15.4%)	21 (12.3%)	479 (17.6%)	451 (17.3%)	28 (16.4%)	534 (19.7%)	493 (19.4%)	41 (24.0%)
	≥45	636 (23.4%)	609 (23.9%)	27 (15.8%)	861 (31.7%)	820 (32.2%)	41 (24.0%)	1049 (38.6%)	1001 (39.4%)	48 (28.1%)
**Duration of employment (years)**										
Mean (SD)		1.4 (0.9)	1.4 (0.9)	1.4 (0.9)	4.4 (0.9)	4.4 (0.9)	4.4 (0.9)	6.4 (0.9)	6.4 (0.9)	6.4 (0.9)
Professional grade										
Managers		284 (10.5%)	251 (9.9%)	33 (19.3%)	285 (10.5%)	252 (9.9%)	33 (19.3%)	291 (10.7%)	256 (10.0%)	35 (20.5%)
Foremen		699 (25.7%)	637 (25.0%)	62 (36.3%)	704 (25.9%)	642 (25.2%)	62 (36.3%)	698 (25.7%)	638 (25.1%)	60 (35.1%)
General labour		1732 (63.8%)	1656 (65.1%)	76 (44.4%)	1726 (63.6%)	1650 (64.9%)	76 (44.9%)	1726 (63.6%)	1650 (64.9%)	76 (44.4%)
**Nature of work**										
Clerical/Administrative		983 (36.2%)	888 (34.9%)	95 (55.6%)	968 (35.7%)	877 (34.5%)	91 (53.2%)	958 (35.3%)	869 (34.2%)	89 (52.0%)
Non-clerical		1732 (63.8%)	1656 (65.1%)	76 (44.4%)	1747 (64.3%)	1667 (65.5%)	80 (46.8%)	1757 (64.7%)	1675 (65.8%)	82 (48.0%)
**History of occupational exposure**										
Yes		596 (21.9%)	556 (21.9%)	40 (23.4%)	596 (21.9%)	556 (21.9%)	40 (23.4%)	596 (21.9%)	556 (21.9%)	40 (23.4%)
No		2119 (78.1%)	1988 (78.1%)	131 (76.6%)	2119 (78.1%)	1988 (78.1%)	131 (76.6%)	2119 (78.1%)	1988 (78.1%)	131 (76.6%)
**Occupational exposure**										
		(N = 596)	(n= 556)	(n = 40)	(N = 596)	(n= 556)	(n = 40)	(N = 596)	(n= 556)	(n = 40)
Noise		118 (19.8%)	114 (20.5%)	4 (10.0%)	118 (19.8%)	114 (20.5%)	4 (10.0%)	118 (19.8%)	114 (20.5%)	4 (10.0%)
Vibration		169 (28.4%)	161 (29.0%)	8 (20.0%)	169 (28.4%)	161 (29.0%)	8 (20.0%)	169 (28.4%)	161 (29.0%)	8 (20.0%)
Inhalation		141 (23.7%)	132 (23.7%)	9 (22.5%)	141 (23.7%)	132 (23.7%)	9 (22.5%)	141 (23.7%)	132 (23.7%)	9 (22.5%)
Chemicals		169 (28.1%)	149 (26.8%)	19 (47.5%)	169 (28.1%)	149 (26.8%)	19 (47.5%)	169 (28.1%)	149 (26.8%)	19 (47.5%)
**Smoking**										
Yes		516 (19.0%)	508 (20.0%)	8 (4.7%)	506 (18.6%)	497 (19.5%)	9 (5.3%)	493 (18.2%)	484 (19.0%)	9 (5.3%)
No		2199 (81.0%)	2036 (80.0%)	163 (95.3%)	2209 (81.4%)	2047 (80.5%)	162 (94.7%)	2222 (81.8%)	2060 (81.0%)	162 (94.7%)
Daily number of cigarettes		(N=516)	(n=508)	(n = 8)	(N = 506)	(n = 497)	(n = 9)	(N = 493)	(n = 484)	(n = 9)
<10		263 (51.0%)	262 (51.6%)	1 (12.5%)	268 (53.0%)	268 (53.9%)	0 (0.0%)	264 (53.6%)	264 (54.6%)	0 (0.0%)
≥10		253 (49.0%)	246 (48.4%)	7 (87.5%)	238 (47.0%)	229 (46.1%)	9 (100.0%)	229 (46.4%)	220 (45.4%)	9 (100.0%)
**Alcohol intake**										
Yes		1089 (40.1%)	1038 (40.8%)	51 (29.8%)	1132 (41.7%)	1076 (42.3%)	56 (32.8%)	1130 (41.6%)	1074 (42.2%)	56 (32.8%)
No		1626 (59.9%)	1556 (59.2%)	120 (70.2%)	1583 (58.3%)	1468 (57.7%)	115 (67.2%)	1585 (58.4%)	1470 (57.8%)	115 (67.2%)
Daily quantity of alcohol		(N=1089)	(n = 1038)	(n = 51)	(N = 1132)	(n = 1076)	(n = 56)	(N = 1130)	(n=1074)	(n = 56)
<4 standard units*		885 (81.2%)	848 (81.7%)	37 (72.6%)	913 (80.7%)	872 (81.0%)	41 (73.2%)	909 (80.4%)	868 (80.8%)	41 (73.2%)
≥4 standard units		204 (18.8%)	190 (18.3%)	14 (27.4%)	219 (19.3%)	204 (19.0%)	15 (26.8%)	221 (19.6%)	206 (19.2%)	15 (26.8%)

* one standard unit = 10ml (~ grams) pure ethanol

**Table 2:** Anthropometric, medical and metabolic characteristics of employees at baseline T0, T3 and T5 medical follow-up periods

Variables	(T0, 2010)			(T3, 2013)			(T5, 2015)		
	Total (N=2715)	Males (n=2544)	Females (n=171)	Total (N=2715)	Males (n=2544)	Females (n=171)	Total (N=2715)	Males (n=2544)	Females (n=171)
**BMI (kg/m2) Mean (SD)**	**22.9 (3.6)**	**22.9 (3.5)**	**24.2 (5.1)**	**24.2 (3.8)**	**24.1 (3.7)**	**25.6 (5.7)**	**24.8 (3.9)**	**24.7 (3.7)**	**26.3 (5.7)**
Underweight (<18.5)	175 (6.5%)	166 (6.5%)	9 (5.2%)	49 (1.8%)	45 (1.7%)	4 (2.3%)	27 (1.0%)	23 (0.9%)	4 (2.3%)
Normal (18.5 – 24.5)	1882 (69.3%)	1778 (69.9%)	104 (60.8%)	1661 (61.2%)	1572 (61.8%)	89 (52.0%)	1495 (55.1%)	1422 (55.9%)	73 (45.0%)
Overweight (25.0 – 29.9)	537 (19.8%)	501 (19.7%)	36 (20.0%)	775 (28.5%)	728 (28.6%)	47 (27.5%)	892 (32.8%)	832 (32.7%)	60 (35.1%)
Moderate Obesity (30.0 – 39.9)	115 (4.2%)	97 (3.8%)	18 (10.5%)	218 (8.0%)	195 (7.7%)	21 (12.3%)	287 (10.6%)	263 (10.3%)	24 (14.0%)
Severe Obesity (≥40.0)	6 (0.2%)	2 (0.1%)	4 (2.3%)	14 (0.5%)	4 (0.2%)	10 (5.8%)	14 (0.5%)	4 (0.2%)	10 (5.6%)
**Fasting glucose (mg/dl)**									
**Mean (SD)**	**93.9 (17.9)**	**93.5 (17.5)**	**99.4 (22.3)**	**98.1 (18.2)**	**97.7 (18.3)**	**104.5 (24.1)**	**100.3 (19.1)**	**99.8 (18.4)**	**107.9 (26.4)**
Hypoglycemia (<70)	61 (2.3%)	57 (2.2%)	4 (2.3%)	16 (0.6%)	14 (0.6%)	2 (1.2%)	4 (0.1%)	3 (0.1%)	1 (0.6%)
Normal (70-100)	2048 (75.4%)	1937 (76.1%)	111 (64.9%)	1783 (65.7%)	1695 (66.6%)	88 (51.5%)	1671 (61.5%)	1590 (62.5%)	81 (47.4%)
Impaired (101-125)	450 (16.6%)	416 (16.4%)	34 (19.9%)	708 (26.1%)	657 (25.8%)	51 (29.8%)	816 (30.1%)	759 (29.8%)	57 (33.3%)
Raised (≥126)	156 (5.7%)	134 (5.3%)	22 (12.9%)	208 (7.6%)	178 (7.0%)	30 (17.5%)	224 (8.3%)	192 (7.6%)	32 (18.7%)
**Total cholesterol (mg/dl)**									
**Mean (SD)**	**118.9 (36.7)**	**118.2 (36.0)**	**128.9 (44.1)**	**132.9 (40.7)**	**132.1 (39.5)**	**145.5 (53.7)**	**135.9 (54.7)**	**135.1 (54.6)**	**147.9 (54.1)**
Normal (<190)	2487 (91.6%)	2341 (92.0%)	146 (85.4%)	2345 (86.4%)	2215 (87.1%)	130 (76.0%)	2328 (85.8%)	2202 (86.5%)	126 (73.7%)
Raised (190-240)	222 (8.2%)	199 (7.8%)	23 (13.4%)	338 (12.4%)	304 (11.9%)	34 (19.9%)	350 (12.9%)	312 (12.3%)	38 (22.2%)
High (>240)	6 (0.2%)	4 (0.2%)	2 (1.2%)	32 (1.2%)	25 (1.0%)	7 (4.1%)	37 (1.4%)	30 (1.2%)	7 (4.9%)
**SBP (mmHg) Mean (SD)**	**124.0 (10.2)**	**124.2 (10.1)**	**120.4 (10.9)**	**130.1 (10.9)**	**130.4 (10.8)**	**126.2 (11.7)**	**132.2 (10.9)**	**132.4 (10.8)**	**128.5 (11.7)**
Normal <120	1183 (43.6%)	1067 (41.9%)	100 (58.5%)	505 (18.6%)	450 (17.7%)	55 (32.1%)	339 (12.5%)	297 (11.6%)	42 (24.6%)
Pre-hypertension (120-139)	1297 (47.8%)	1255 (49.3%)	51 (22.8%)	1674 (61.7%)	1588 (62.4%)	86 (50.3%)	1726 (63.6%)	1638 (64.4%)	88 (51.4%)
Stage1 Hypertension (140-159)	226 (8.3%)	213 (8.4%)	20 (11.7%)	517 (19.0%)	488 (19.2%)	29 (17.0%)	627 (23.1%)	587 (23.1%)	40 (23.4%)
Stage2 Hypertension (160-179)	9 (0.3%)	9 (0.4%)	0 (0.0%)	19 (0.7%)	18 (0.7%)	1 (0.6%)	23 (0.8%)	22 (0.9%)	1 (0.6%)
**DBP (mmHg) Mean (SD)**	**79.2 (8.3)**	**79.2 (8.4)**	**79.4 (8.3)**	**82.1 (8.5)**	**82.1 (8.5)**	**82.7 (8.3)**	**83.9 (8.2)**	**83.8 (8.2)**	**84.5 (8.3)**
Normal (<80)	1550 (57.1%)	1450 (57.0%)	116 (67.8%)	1079 (39.7%)	1013 (39.8%)	66 (38.6%)	803 (29.6%)	755 (29.7%)	48 (28.1%)
Pre-hypertension (80-89)	890 (32.8%)	839 (33.0%)	42 (24.6%)	1202 (44.3%)	1132 (44.5%)	70 (40.9%)	1370 (50.5%)	1287 (50.6%)	83 (48.5%)
Stage1 Hypertension (90-99)	261 (9.6%)	241 (9.5%)	13 (7.6%)	398 (14.7%)	363 (14.3%)	35 (20.5%)	476 (17.5%)	438 (17.2%)	38 (22.2%)
Stage2 Hypertension (100-109)	14 (0.5%)	14 (0.5%)	0 (0.0%)	36 (1.3%)	36 (1.4%)	0 (0.0%)	66 (2.4%)	64 (2.5%)	2 (1.2%)

**Figure 2:**
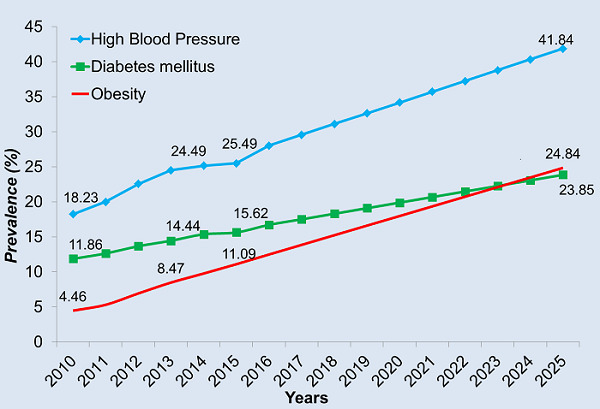
Prevalence of ODH for 6 years follow-up following baseline measures with projection on consequent morbidity over the subsequent 10 years without preventative intervention measures

Longitudinal changes in metabolic and anthropometric risk factors for ODH is presented in Table 3. Using repeated paired t-tests, significant (p < 0.001) increases in mean total blood cholesterol, fasting blood glucose, SBP, DBP, and BMI was recorded. Figure 2 shows trends for ODH prevalence during the 5 years post-baseline measures and projected increases of NCD-related t morbidity over the subsequent 10 years (2015 to 2025) if left unchecked. There was a highly significant increase in the prevalence of ODH from baseline to both follow-up point (x^2^ test p < 0.001). The projection model indicates increases in ODH in a 10-yr period equivalent of 25% obesity, 24% diabetes, and 42% hypertension, provided intervention programs were not introduced or completely ineffective. Table 4 presents the association between selected behavioral risk factors and occurrence of ODH during the observation period. Reported alcohol intake increased the occurrence of obesity risk by 1.5-fold (p < 0.005) and hypertension 2.5-fold (p < 0.0001) compared to those non-drinkers, while for diabetes there was a minimal association with increased risk. When the intake frequency of ethanol exceeded four standard units per day, the occurrence of ODH increased 2.1 (p < 0.0001), 2.4 (p < 0.005), and 3.3 (p < 0.0001) times, respectively compared to those drinking less than 4 units per day. Smoking decreased risk of obesity and diabetes (RR=0.34; p < .005) and 0.81 (p < 0.0001), respectively, while increasing risk of hypertension over 4-fold (RR=4.06; p < 0.0001) compared to non-smokers. Lastly, for those who smoked tobacco, the daily use of 10 or more cigarettes was associated with reduced obesity (RR=0.21; p < 0.05), while increasing risk of diabetes (RR=3.72; p < 0.05) and hypertension (RR=5.17; p < 0.0001). The relationship between occupational exposures/ hazards and occurrence of ODH over time is shown in Table 5. The occurrence of hypertension in study subjects who reported exposure to work-related vibration was 4.9 times higher than in those not exposed, while the risk of becoming obese had a negative correlation (0.88; p < 0.0001)). Reported exposure to excess noise increased the risk of hypertension 5.32 times (p < 0.0001) compared to those working in less noisy environments. With exposure to industrial chemical agents, the risk of obesity, diabetes, or hypertension was respectively 2.06 , 4.9, and 1.56 times (p < 0.0001) greater than those not working in the presence of chemicals. Lastly, with regard to the type of employment, in general categorical terms, office-based workers were 2.72 times more likely to be obese, 1.98 times at greater risk of becoming diabetic, and 1.93 times significantly (p < 0.0001) more likely to be hypertensive compared to field-based employees.

**Table 3:** Comparison statistics for changes in metabolic and anthropometric risk factors at baseline, year-3 and year-5 follow-up of 2,715 employees

Risk factor	Baseline (T0)	T3 (T3)	T5-Year (T5)
**Serum total cholesterol (mg/dl)**			
Mean (SD)	118.9 (36.7)	132.9 (40.7)	135.9 (54.7)
Mean (SE) Δ change from T0		+14.1(0.3)	+17.0 (0.3)
95% CI for Δ change		[13.4 - 14.7] *	[15.5 - 18.6] *
**Fasting plasma glucose (mg/dl)**			
Mean (SD)	93.9 (17.9)	98.1 (18.2)	100.3 (19.1)
Mean (SE) Δ change from T0		+4.2 (0.1)	+6.5 (0.1)
95% CI for Δ change		[4.1 - 4.5] *	[6.2 - 6.7] *
**Systolic blood pressure (mm Hg)**			
Mean (SD)	124.0 (10.2)	130.1 (10.9)	132.2 (10.9)
Mean (SE) Δ change from T0		+ 5.3 (0.1)	+8.2 (0.1)
95% CI for Δ change		[5.9 - 6.3] *	[7.9 - 8.4] *
**Diastolic blood pressure (mm Hg)**			
Mean (SD)	79.2 (8.3)	82.1 (8.3)	83.9 (8.2)
Mean (SE) Δ change from T0		+2.9 (0.4)	+4.6 (0.4)
95% CI for Δ change		[2.1 - 3.8] *	[4.5 - 5.8] *
**Body mass index (kg/m^2^)**			
Mean (SD)	22.9 (3.6)	24.1 (3.9)	24.8 (3.9)
Mean (SE) Δ change from T0		+ 1.2 (0.2)	+ 1.9 (0.2)
95% CI for Δ change		[1.1 - 1.3] *	[1.8 - 1.9] *

p-values are based on repeated paired *t*-test statistic

* statistical significance set at *p*<0.001

**Table 4:** Behavioral risk factors and risk of ODH occurrence from baseline and consecutive 5-year follow-up

Risk Factors	Condition								
	Obesity			Diabetes mellitus			High Blood Pressure		
	Total (N=2594)	Present (n=180)	RR [IC95%]	Total (N=2393)	Present (n=102)	RR [IC95%]	Total (N=2220)	Present (n=197)	RR [IC95%]
**Alcohol intake**									
Yes	1050	91 (8.67%)	1.50 [1.13 - 1.99]	990	43 (4.34%)	1.03 [0.70 - 1.51]	953	128 (13.43%)	2.47 [1.86 – 3. 26]
No	1544	89 (5.76%)		1403	59 (4.20%)		1267	69 (5.44%)	
Daily quantity of alcohol	(n=1050)			(n=990)			(n=953)		
≥ 4 standard units	191	29 (15.18%)	2.10 [1.39 - 3.18]	178	15 (8.43%)	2.36 [1.29 - 4.31]	186	48 (25.81%)	3.30 [2.34 - 4.65]
< 4 standard units	859	62 (7.21%)		813	29 (3.57%)		767	60 (7.82%)	
**Smoking**									
Yes	488	13 (2.66%)	0.34 [0.19 - 0.58]	435	12 (2.76%)	0.81 [0.45 - 1.50]	479	104 (21.71%)	4.06 [3.13 - 5.28]
No	2106	167 (7.93%)		1958	66 (3.37%)		1741	93 (5.34%)	
Daily number of cigarettes	(n=488)			(n= 435)			(n=479)		
≥10	228	2 (0.88%)	0.21 [0.05 - 0.92]	152	8 (5.26%)	3.72 [1.14 - 12.2]	247	88 (35.62%)	5.17 [3.13 - 8.53]
< 10	260	11 (4.23%)		283	4 (1.41%)		232	16 (6.90%)	

RR = Relative Risk between dichotomous responses

**Table 5:** Occupational exposures and risk of ODH occurrence from baseline and consecutive 5-year follow-up

Exposure				Condition					
	Obesity			Diabetes mellitus			Hypertension		
	Total (N=2594)	Present (n=180)	RR [IC95%]	Total (N=2393)	Present (n=102)	RR [IC95%]	Total (N=2220)	Present (n=197)	RR [IC95%]
**Vibration**									
Yes	163	10 (6.13%)	0.88 [0.47 - 1.63]	141	9 (6.38%)	1.54 [0.80 - 3.00]	75	26 (34.66%)	4.90 [3.47 - 6.90]
No	2431	170 (7.00%)		2252	93 (4.13%)		2415	171 (7.97%)	
**Noise**									
Yes	113	12 (10.62%)	1.57 [0.90 - 2.73]	103	4 (3.88%)	0.91 [0.34 - 2.41]	81	33 (40.74%)	5.32 [3.93 - 7.18]
No	2481	168 (6.77%)		2290	98 (4.28%)		2139	164 (7.66%)	
**Chemical**									
Yes	281	36 (12.81%)	2.06 [1.46 - 2.90]	240	36 (15.00%)	4.90 [3.33 - 7.18]	246	32 (13.00%)	1.55 [1.10 - 2.21]
No	2313	144 (6.22%)		2153	66 (3.06%)		1974	165 (8.36%)	
**Type of work**									
Clerical	922	108 (11.71%)	2.72 [2.04 - 3.62]	845	53 (6.27%)	1.98 [1.35 - 2.90]	753	98 (13.01%)	1.93 [1.48 - 2.51]
No-clerical	1672	72 (4.31%)		1548	49 (3.16%)		1467	99 (6.75%)	

RR = Relative Risk between dichotomous responses

While the relative risk for ODH between males and females were similar over time, there were some notable differences between male and female employees regards measured risk factors and outcomes from baseline to T5 (Table 1, Table 2). Mean age between genders were similar as well as duration of employment with the mine. Throughout the observation period, both reported similar percentages of occupational exposure, while females were more likely (p < 0.0001) to be in clerical/administrative positions than males. Overall, females were far less likely to smoke and drink alcohol than their male counterparts. Mean number of subjects reporting smoking and alcohol consumption (yes/no) were significantly greater (p < 0.0001 and p = 0.01, respectively) in males than females across the three observation times. However, those females who did report smoking and alcohol use were more likely by percentage to smoke or consume more on a daily basis than males. For those reporting alcohol use, within gender there was no difference (p=0.14) in daily quantity consumed throughout the observation period. Regards ODH outcome variables, females were more likely to have a greater BMI, hyperglycemic, and elevated cholesterol; whereas hypertensive state was generally higher in males. Using Fisher's exact test to compare males and females at baseline and T5, significant differences (p < 0.006 to 0.0001) were noted in BMI (combined “overweight", “moderate” and “severe” obesity categories), fasting glucose (combined “impaired” and “raised"), total cholesterol (combined “raised” and “high"), SBP and DBP (combined “pre-hypertension", “stage 1” and “stage 2” hypertension, respectively). The lone exception was for DBP at T5 (p = 0.73) indicating no differences between gender. Within gender, increases for all outcome variables between T0 and T5 ranged from moderate (p < 0.01) for elevated cholesterol in females to highly significant (p < 0.0004 - 0.0001) for all other categories. In all cases, both males and females had increased rates of relative risk from baseline to T5.

## Discussion

The objective of this study was to measure trends in ODH prevalence over time and to identify important risk factors contributing to changes in prevalence in the study population. This study follows a published baseline cross-sectional study describing the point prevalence of ODH and associated risk factors in the same mining employee population derived from occupational health information recorded in 2010 [14]. The current study covers socio-demographic, medical and occupational information of individual employees selected (study eligible) from the general mine workforce population required to undergo annual OMCU on pre-employment or follow-up medicals over a period of 5 consecutive years from 2011 to 2015 (time T1-T5), with 2010 serving as “baseline” (time T0). Using this set baseline, subsequent annual OMCUs allowed longitudinal measurement of trends in ODH prevalence and identification of important risk factors contributing to changes in ODH prevalence in the study cohort. To achieve this, three benchmark time periods of OMCU data were collated: baseline (T0, 2010), year three (T3, 2013), and year five follow-up (T5, 2015). Baseline was either the initial medical screening of a new employee or a routine medical check-up. Mean values of primary quantitative risk indicators (BMI, fasting glucose, total cholesterol, SBP and DBP) were recorded. The total number of accessible files included in the initial analysis (data not presented herein) was T0: 2,749, T3: 4,751, and T5: 5,015. Based on study inclusion criteria to conduct a longitudinal assessment, 2,715 individuals who had complete medical screening records between 2010 to 2015 where registered into the study cohort.

Study findings clearly indicated that ODH constitutes a major and alarming health challenge for the workforce population. The burden of ODH-related non-communicable diseases is high and is predicted to increase in the years to come if interventions are not put in place. Results from the cross-sectional study conducted in the same general workforce mine population defined the burden of ODH and key risk factors in 2010 [14]. Like another study on office workers in Kinshasa [19], the present study showed that mine workforce is also a high-risk occupational group for NCDs associated with high prevalence of ODH risk factors. Without intervention, the percentage of pre-obese (19.7%), pre-diabetic (16.5%), and pre-hypertensive (47.8%) individuals identified in the 2010 baseline study are predicted to transition to full ODH status eventually, thus increasing the burden of NCDs on workforce productivity and society as a whole [14]. Longitudinal analysis showed that the prevalence of ODH and mean quantitative health risk markers (fasting glucose, total cholesterol, BMI, SBP, DBP) increased significantly between 2010 and 2015. This is similar to a longitudinal study conducted in a large mining work force in Indonesia showing a marked increase in the prevalence of NCDs and their risk factors over time [20]. These results corroborate WHO´s estimates and conclusions from recent studies that show clearly the burden of NCDs is increasing rapidly throughout the world regardless of income and country development status; and that the largest portion of this alarming rise is occurring in Africa [1-4,6,18,21].

In our study cohort, between 2010 and 2015, the prevalence of obesity increased by 148.6% (4.46% to 11.09%); 31.7% for the diabetes (11.86% to 15.62%), and 39.8% for hypertension (18.23% to 26.49%). These unmistakable trends are far higher than global trends with increases of 18.9% and 7% for obesity and diabetes, respectively, between 2010-2014 [4]. Between 2000 and 2010, the burden of disease attributable to hypertension worldwide increased by 55% [10,11], while a 170% increase was recorded in a 20 years period (1986 - 2006) in Kinshasa, DRC [22,23]. Based on our projections, in the next ten years (2015 - 2025) minus effective intervention programs, the prevalence of obesity will increase approximately 127% (11% to 25%); 50% for diabetes (16% to 24%), and 62% for hypertension (26% to 42%). This dramatic upward trend may hinder the goal of 25x25 (25% reduction of NCD mortality by 2025) modelling target [24]. In the current study, the projected increase in prevalence of diabetes from 2015 to 2025 (50%), is far higher than that predicted to occur in the overall DRC between 2013 and 2035 (15%) [25]. Additional to the presence of metabolic, behavioral and occupational risk factors the increased prevalence of ODH in the studied population may, in part, be age-related (consequent aging of the cohort over time), and the high baseline proportion of pre-obese, pre-diabetic and pre-hypertensive individuals. The prevalence of hyperglycemia between 2010, and 2015 was higher than reported by WHO using the same benchmarks, for the general population of the DRC [26]; and among mining employees in Indonesia [20]. By comparison, mean values of fasting glucose and SBP for the 3 times intervals were also higher than those reported in Indonesia [20]. Mean total blood cholesterol and BMI found in our DRC observations were lower than those reported in Indonesia, but higher than for the general population of the DRC [4,20]. Findings from our study also show that mean values of Δ, measuring change from baseline for BMI were higher and more rapid than those in Indonesia [20]. Although the prevalence of tobacco reported use remained stable at around 20%, there was a significant increase in the proportion of individuals who smoked more than 10 cigarettes per day. The proportion of individuals who reported alcohol use during the investigation period remained stable at around 40%, while showing a slight increase in the number of those indicating 4 or more standard units of alcohol consumed daily (18.8% in 2010 and 19.6% in 2015). The introduction of a mandatory random blood alcohol check in 2013 by the mining company appears to have had no impact on overall alcohol consumption in general.

Over time, there was a statistically significant decrease in the proportion of underweight and normal weight individuals, in favor of overweight and obesity. Similarly, an increase in the proportions of pre-diabetic/diabetic, and pre-hypertensive/ hypertensive individuals, was observed. These longitudinal trends are suggestive of a general transition in the workforce lifestyle and behavior. Indeed, this population consists largely of people who have lived for long periods in a different environment before migrating to the mine site for work and a steady income. In recent decades, more traditional societies in many developing countries have experienced rapid and poorly planned urbanization and population growth, leading to vastly contrasting lifestyles characterized by inadequate or poor nutrition, reduced physical activity, increased tobacco and alcohol consumption, all contributing to the increase in the prevalence of NCDs [21,24,27]. Several studies have shown that individuals living in communities that are undergoing rapid development are also at higher risk for developing NCDs. Some drivers of development, such as the greater availability of transportation, the higher consumption of processed and imported (non-traditional) foods, reliance on industrial agricultural products, more sedentary lifestyles, inadequate diet and sleep, increased alcohol and tobacco use, linked to increase risk of NCDs [1,4,9,15,27].

Compared to other occupational sectors, mine workers have been identified as having a higher risk of chronic health problems [28]. These can be caused by exposure to a wide range of factors and hazards including physical (high altitude, noise, vibration, irradiation, intense heat), chemicals (air pollution, silica dust, hydrocarbons, industrial solvents, acids, paints, etc.), biological; ergonomic, psychosocial (e.g., isolated work, night rotations, high stress, alcohol abuse, excess smoking), and nutritional (e.g., catered food service leading to average higher caloric intake, increased, consumption of processed food) that are common to mining sites and may influence hypercholesterolemia, hyperglycemia, hypertension and increased BMI [28-33]. In Norway, an association between frequent use of staff canteens, unhealthy diet (food selection) and obesity was reported in adults [34]. In our observations, the risk of obesity increased with use of ≥ 4 standard units of ethanol per day, having a clerical/ administrative work position (i.e., more sedentary employment), and exposure to various chemicals; whereas obesity rates were lower with more exposure to vibration and daily use of ≥ 10. Others have reported a negative correlation between tobacco use and body weight; smokers, especially more frequent tobacco users, are at lower risk of becoming obese, while cessation of smoking led to an increase in BMI [35-39]. The negative correlation between smoking and BMI may be the result of reduced appetite caused by nicotine [40], and that smoking impairs normal taste and olfactory functions that can suppress impact dietary intake [41,42]. The risk of diabetes occurrence in our study cohort increased with daily quantity of alcohol and number of cigarettes smoked, having a clerical/ administrative position, and frequent exposure to chemicals. Alcohol abuse may increase the risk of diabetes by inducing insulin resistance [43]. The mechanisms by which greater tobacco use, as well as occupational exposure to chemicals (excluding or additional to cigarettes), favor the occurrence of diabetes are unclear. However, office-based work, by circumstance and compared to non-clerical positions can promote more sedentary activity, thereby increasing the risk of obesity, thus diabetes. The risk of becoming hypertensive was higher among those consuming ≥ 4 standard units of alcohol daily. Several mechanisms have been implicated, primarily the hypersecretion of catecholamines in the central nervous system as well as kidneys and adrenals, cortisol, plasma angiotensin and aldosterone due to increased plasma renin activity. Additionally, excessive chronic ethanol consumption increases water and sodium retention, a hypertrophy of the vascular smooth muscle wall, and an increase in cytoplasmic calcium that is responsible for vasoconstriction and therefore an increase in blood pressure [43].

Smoking, especially 10 or more cigarettes daily, increased the risk of hypertension in our study population. In China, the risk of hypertension in adults increased with the use of ≥ 15 cigarettes per day [44]. Tobacco is a well-known major risk factor for NCDs [11] and its role in the occurrence of hypertension and CVD is well established [45-47]. Study employees in clerical (office-based) work positions were at a higher risk of hypertension than those working in field-based environments (e.g., workshops, construction sites, etc.). Greater mobility and physical activity reduce the risk of hypertension compared to more sedentary forms of work. Interventional programs and promotion of behavioral changes to increase physical activities in the office setting would help to alleviate this insidious risk factor. Regular or excessive exposure to chemicals (industrial solvents, acids, bases, sulfur, paints, hydrocarbons) and excessive noise and vibration in the workplace were associated with increased the risk of hypertension in our study cohort. Chemicals can induce toxic-related hypertension, aliphatic amines, polycyclic hydrocarbons, carbon disulphide, and heavy metals are believed to act primarily on blood vessels via calcium channel blockade and interaction with intracellular proteins of the vascular wall [48]. Adults exposed to road traffic and aircraft noise show an association to higher prevalence of hypertension compared to unexposed populations [49,50]. Noise appears an important risk factor for hypertension and CVD therefore encouraging people working in noisy environments to routinely wear hearing protection equipment.

This study presents some limitations for deriving generalizations to the overall population in the DRC. The first is the predominance of males (93.7%) in our study cohort, a direct reflection of the gender distribution of the mine workforce. The low proportion of females (6.3%), while wage earning and better educated in general, makes difficult any accurate representation and extrapolation to the general female population. The second limitation is that information on alcohol use and smoking was based on an individual's recall and willingness to provide accurate information, thus raising concerns for potential response bias. Finally, working with archived data collected by different people over time presents potential measurement, observation or information bias.

## Conclusion

Obesity, diabetes and hypertension are early manifestations of the growing burden of NCDs in the TFM workforce. The prevalence of each condition is high and increasing. The high proportion of pre-obese, pre-diabetic and pre-hypertensive states, the upward trend in mean values of quantitative risk indicators - fasting glucose, blood cholesterol, BMI, blood pressure readings - over the observation period, including projections for the next decade, predict a significant increase in the burden of ODH in the coming years, this barring the implementation of a comprehensive prevention program. The progressive increase in debilitating, chronic NCDs will likely have a significant financial impact on the company’s productivity (absenteeism, loss work time, etc.) and decrease profitability as the workforce struggles to combat health issues due to poor medical management of chronic disease conditions and lack of lifestyle changes (e.g., diet, exercise, etc.). Combined, ODH contributes to significant CVD and neurovascular diseases due to irreparable damage to blood vessels and target organs including heart, brain and kidneys. This will inevitably generate excess expenses for largely preventable or manageable medical conditions. The resulting early onset morbidities and mortality places tremendous socio-economic burdens on workforce families and communities. The paucity of detailed information on the African workforce is both revealing of the general neglect on NCDs on the continent and the significant challenges ahead to combat it. This study paves the way for further research on the consequences of NCDs on the mine workforce productivity and broader assessments of targeted interventions to help reduce the health burden to individuals and wider impact on communities.

### What is known about this topic

- The World Health Organization (WHO) identified hypertension as the greatest NCD problem in the Democratic Republic of the Congo (DRC) in 2014, with one of the highest prevalence rates in Africa (24.8% of adults);
- From 2010 to 2014, the prevalence of overweight in adults aged 18 and over in the DRC increased from 18.8 to 20.6%, while obesity rates rose from 3.7 to 4.4%;
- In the DRC, diabetes mellitus has increased steadily from 5.7% to 6.1% between 2010 and 2014; whereas 11.7% of the TFM workforce was clinically diabetic in 2010.

### What this study adds

- Obesity, diabetes and hypertension are early manifestations of the growing burden of NCDs in the TFM workforce. The prevalence of each condition is high and increasing. Between 2010 and 2015, prevalence increased from 4.5% to 11.1% for obesity, 11.9% to 15.6% for diabetes, and 18.2% to 26.5% for hypertension;
- The projection model indicates increases in ODH in a 10-yr period equivalent of 25% obesity, 24% diabetes, and 42% hypertension, provided intervention programs were not introduced or completely ineffective.

## Competing interests

The authors declare no competing interests.
